# CAR T Cell Locomotion in Solid Tumor Microenvironment

**DOI:** 10.3390/cells11121974

**Published:** 2022-06-20

**Authors:** Duy T. Nguyen, Elizabeth Ogando-Rivas, Ruixuan Liu, Theodore Wang, Jacob Rubin, Linchun Jin, Haipeng Tao, William W. Sawyer, Hector R. Mendez-Gomez, Matthew Cascio, Duane A. Mitchell, Jianping Huang, W. Gregory Sawyer, Elias J. Sayour, Paul Castillo

**Affiliations:** 1 Department of Mechanical and Aerospace Engineering, University of Florida, Gainesville, FL 32611, USA; nguyenduy2308@ufl.edu (D.T.N.); wsawyer@ufl.edu (W.W.S.); wgsawyer@ufl.edu (W.G.S.); 2 Department of Neurosurgery, University of Florida, Gainesville, FL 32608, USA; elizabeth.ogando-rivas@neurosurgery.ufl.edu (E.O.-R.); ruixuan.liu@neurosurgery.ufl.edu (R.L.); linchun.jin@neurosurgery.ufl.edu (L.J.); haipeng.tao@neurosurgery.ufl.edu (H.T.); hector.mendezgomez@neurosurgery.ufl.edu (H.R.M.-G.); duane.mitchell@neurosurgery.ufl.edu (D.A.M.); jianping.huang@neurosurgery.ufl.edu (J.H.); elias.sayour@neurosurgery.ufl.edu (E.J.S.); 3 College of Medicine, University of Florida, Gainesville, FL 32610, USA; teddy6502@ufl.edu; 4 Warrington College of Business, University of Florida, Gainesville, FL 32610, USA; j.rubin@ufl.edu; 5 Department of Pediatrics, Division of Pediatric Hematology Oncology, University of Florida, Gainesville, FL 32610, USA; mcascio@ufl.edu

**Keywords:** CAR T cells, solid tumors, T cell migration, trafficking, tumor microenvironment, immunotherapy, adoptive T cell therapy, 3D in vitro models

## Abstract

The promising outcomes of chimeric antigen receptor (CAR) T cell therapy in hematologic malignancies potentiates its capability in the fight against many cancers. Nevertheless, this immunotherapy modality needs significant improvements for the treatment of solid tumors. Researchers have incrementally identified limitations and constantly pursued better CAR designs. However, even if CAR T cells are armed with optimal killer functions, they must overcome and survive suppressive barriers imposed by the tumor microenvironment (TME). In this review, we will discuss in detail the important role of TME in CAR T cell trafficking and how the intrinsic barriers contribute to an immunosuppressive phenotype and cancer progression. It is of critical importance that preclinical models can closely recapitulate the in vivo TME to better predict CAR T activity. Animal models have contributed immensely to our understanding of human diseases, but the intensive care for the animals and unreliable representation of human biology suggest in vivo models cannot be the sole approach to CAR T cell therapy. On the other hand, in vitro models for CAR T cytotoxic assessment offer valuable insights to mechanistic studies at the single cell level, but they often lack in vivo complexities, inter-individual heterogeneity, or physiologically relevant spatial dimension. Understanding the advantages and limitations of preclinical models and their applications would enable more reliable prediction of better clinical outcomes.

## 1. Introduction

Despite the great progress in understanding the biology of cancer and the discovery of new targeted therapies, cancer continues to be the second leading cause of mortality in the US with 1.8 million new cancer cases diagnosed in 2020 and over 600,000 cancer deaths in the United States [1]. The immune system is our body’s natural defense against predation which includes the development of cancer cells [2,3,4]. Unfortunately, cancer cells are often able to avoid immune destruction [5], leading to tumor formation. T cell immunotherapy has proven effective in restoring immune response against a plethora of malignancies [6] but remains limited by a dearth of antigenic targets. To overcome such obstacles, chimeric antigen receptor (CAR) T cells are designed to fuse the specificity of single-chain variable fragment regions from an antibody to the specific and cytolytic function of T cells, broadening the applicability of this new technology to surface antigen targets. Thus, CAR T cell therapy has emerged as a promising immuno-oncology approach that could enhance target recognition and cytotoxic function against antigen-specific cancer cells without the need for major histocompatibility complex and T cell receptor interactions. CAR T cells from patient-derived lymphocytes are genetically engineered to express an extracellular antibody fragment linked to modified intracellular co-stimulatory domains that induce activation upon engagement to the target antigen [7,8,9] and have led to almost 95% complete remission rates at 1 month post-infusion in patients with B cell leukemias and up to 40% in patients with B cell lymphomas [8]. However, the results have not been translated to solid tumors largely due to major challenges including on-target off-tumor toxicity, cytokine release syndrome, tumor heterogeneity, and poor CAR T trafficking into the immunosuppressive tumor microenvironment (TME).

In this review, we aim to discuss barriers to CAR T trafficking and locomotion within the immunosuppressive TME. The ability of CAR T cells to efficiently migrate to the tumor site, infiltrate suppressive barriers, and survive the harsh TME represents a crucial prerequisite for carrying out the anti-tumor function. To address this important challenge, preclinical models must closely recapitulate the spatial and temporal dynamics of a TME which imposes physical and biochemical barriers against CAR T trafficking and fitness. We will also discuss various preclinical models and platforms that have been employed to study CAR T infiltration and killing of solid tumors [10]. In particular, the complex TME of solid tumors highlights the need for three-dimensional (3D) in vitro models to better study cell–cell and cell–extracellular matrix (ECM) interactions. We discuss how CAR T cells can be engineered to eradicate antigen-specific cancer cells that are heterogeneously expressed, evolving, and shielded by layers of biochemical and physical barriers using novel 3D in vitro models and platforms [11,12,13].

## 2. CAR T Cell Trafficking and Infiltration

### 2.1. The Immunosuppressive Tumor Microenvironment and Physical Barriers Favorable to Resilient Tumor Growth

**Figure 1:**
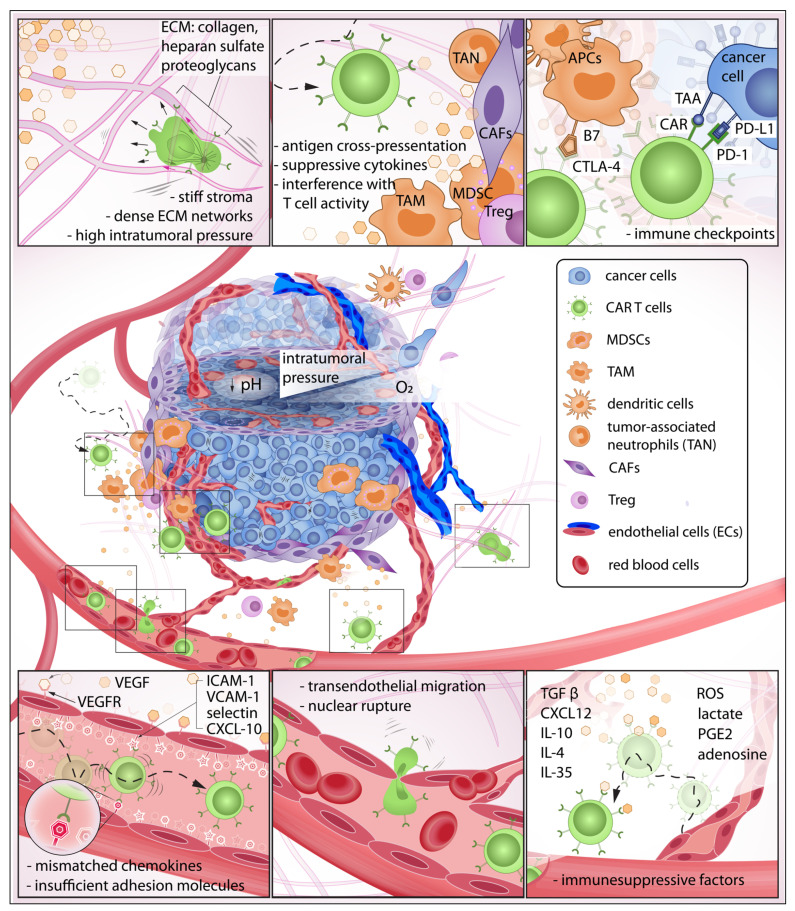
The exhausting journey of CAR T cells in the TME: In this journey, CAR T cells must be able to detect chemokines at the tumor site, effectively roll and adhere to the blood vessel wall, initiate transendothelial migration, and invade the tumor stroma. Here, the immune cells must overcome various biochemical and physical barriers to then encounter pro-tumor cells that suppress CAR T cell activity. Some CAR T cells may eventually make contact with target cancer cells that express abundant immune checkpoints, further reducing anti-tumor function.

Circulating CAR T cells must gain sufficient traction and adhesion against receptors on the endothelial wall to initiate extravasation (Figure 1). However, secretion of angiogenic factors VEGF and bFGF during tumorigenesis leads to insufficient expression of adhesion molecules—such as intercellular adhesion molecule 1 (ICAM-1), 2 (ICAM-2), and vascular cell adhesion molecule 1 (VCAM-1)—on endothelial cells which prevents efficient T cell engagement [14]. Adhesion molecules expressed on both immune cells and endothelial walls modulate interactions necessary for transendothelial migration (TEM). Although mechanisms of TEM have been reported extensively, identification of a comprehensible approach to enhance the extravasation of T cells remains elusive. Instead, T cells have been engineered to target tumor-associated endothelial receptors such as VEGFR2 to disrupt tumor-supporting vasculature, enhance T cell trafficking, and deprive the tumor of nutrient and oxygen supplies [15,16]. This approach revealed the efficient killing of in vitro tumor spheroids [16] and prolonged survival in animal models [15,17,18].

**Figure 2:**
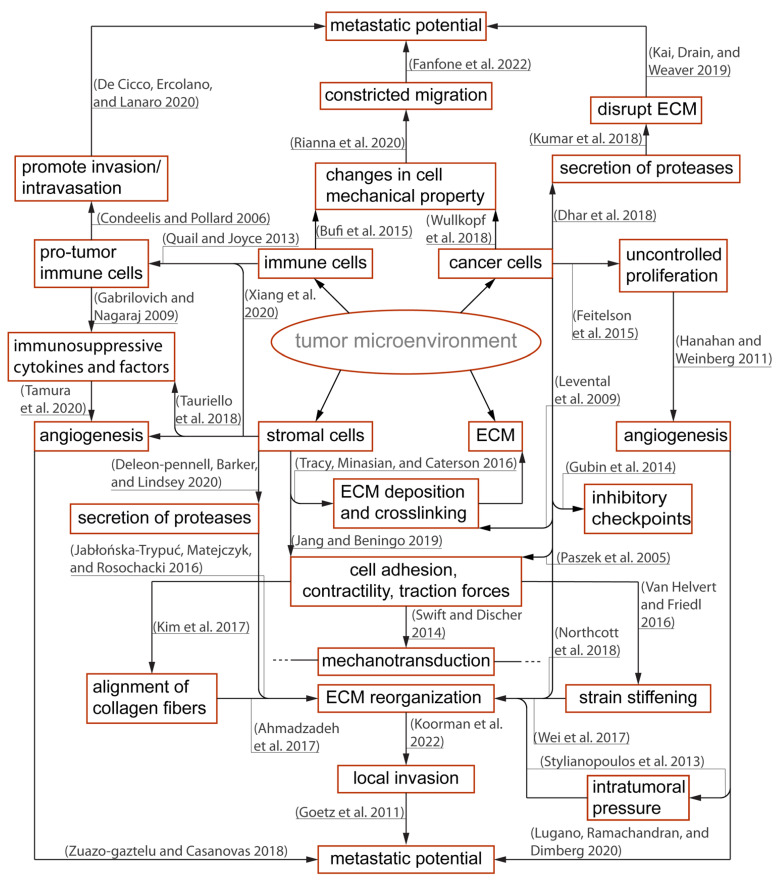
Overview of the immunosuppressive tumor microenvironment (TME) and important contributors to tumor progression: Within the TME, there is a dynamic relationship between peritumoral and intratumoral components that include immune cells, cancer cells, and the stromal elements often exhibiting context-dependent functionality. Different components of the TME carry out pro- or anti-tumor functions, and such polarizations are caused by reciprocal interactions with physical and biochemical cues from the surroundings. The polarized functions are not distinctive but rather heterogenous contributing to an immunosuppressive TME [5,19,20,28,29,35,39,40,46,48,53,54,55,56,57,58,59,60,61,62,63,64,65,66,67,68,69,70,71,72,73,74,75].

CAR T cells must traverse the endothelial junction and navigate through the tumor stroma that is inherently armed with abundant immunosuppressive factors to reach target cancer cells. The TME has long been identified as an active contributor to cancer progression [5]. In this neoplastic microenvironment, different cell types interact with each other and the surrounding ECM to orchestrate a constantly evolving habitat that is favorable to resilient tumor growth [19,20,21] and deleterious to immune function [22,23,24,25] (Figure 2). For instance, CAR T cells—upon successful infiltration of the TME—demonstrated a rapid loss of function [26]. Infiltrating CAR T isolated from xenograft tumors and cultured in vitro for 24 h regained killing ability superior to those freshly isolated [26]. This emphasizes the immunosuppressive influences of the TME that suppress CAR T anti-tumor activity. Furthermore, the stromal, immune, and cancer cells constantly remodel the TME in response to the surrounding biochemical and biophysical cues [19,27,28,29,30] (Figure 2). Unlike hematologic malignancies—which can be freely accessed by CAR T cells—solid tumors have a complex three-dimensional structure wherein malignant cells are more difficult to access [31,32,33,34]. One hallmark of tumor progression is the elevated stiffening of the ECM due to increased matrix crosslinking, collagen deposition, and fiber alignment which regulate cell migration, proliferation, and apoptosis via mechanotransduction [35,36,37,38,39]. The stiffening effect is often caused by collagen deposition and crosslinking, mediated by increased secretion of lysyl oxidase (LOX) [30,40] and overproduction of other ECM components such as heparan sulfate proteoglycans (HSPGs) [41,42]. Due to this physical constraint, primary tumor growth and cell migration within the TME are greatly dependent on matrix metalloproteinases (MMP) and heparinase enzymes to degrade and reorganize the crosslinked networks [43,44,45,46,47,48]. However, T cells do not often secrete enzymes to degrade ECM, but rather choose the path of least resistance [49] or follow contact guidance imposed by architectural features of the surrounding ECM [50,51]. Therefore, densely packed and oriented stromal fibers have been reported to impose significant challenges to T cell infiltration [52].

Tumor growth is critically dependent on angiogenesis [27,76,77] which is promoted in an MMP–dependent manner [43]. Given nutrient support from neovessel networks, the growing tumor continues to modify its microenvironment through elevation of growth-induced solid stress [78,79], interstitial fluid pressure [80,81], enzymatic secretion [40,43], and cellular contractility onto the ECM [68,71]. One example of ECM modification is the physical alignment of collagen fibers due to cell-induced strain stiffening [28,69,82] from cancer cells and their stromal neighbors such as cancer-associated fibroblasts (CAFs) [28,64,67]. This reorganization further stiffens the TME, suppresses immune activity and mobility [83,84], and promotes local invasion of cancer cells along the aligned fibers [55,73,83,85,86,87,88]. In addition, intratumoral pressure caused by growth-induced solid stress and interstitial fluid pressure obstruct delivery of therapeutic agents and trafficking of immune cells to the solid tumors [89]. Elevated interstitial fluid pressure—reported in most solid cancers [90]—is mainly caused by leaky blood vessels, impaired lymphatic transport, and hyaluronan swelling [72,91]. The fluid pressure contributes to an increasing growth-induced solid stress and presents a non-trivial barrier to immune infiltration and uptake of therapeutic agents [90,92]. An in vitro study demonstrated that an interstitial fluid pressure ≥ 1 KPa—simulated by hydrostatic pressure—is sufficient to physically hindered antigen-specific T cell infiltration into tumor site [93]. Furthermore, growth-induced solid stress (≥2 kPa) —while impeding proliferation of cancer cells [78,94] —can collapse blood vessels [72] and contribute to vasculature abnormalities and subsequent increase in interstitial pressure [71]. The reciprocal relationship further escalates total intratumoral pressure, exacerbates hypoxia, and increases pH and metabolic waste accumulation. TME determinants—such as hypoxia [95] and TGFβ [63]—stimulate the production of ECM [23,65,96,97] and LOX [98] to further reinforce the physical barriers that impede CAR T infiltration [33,99]. The feedback loop continually contributes an immunosuppressive TME responsible for tumor pathogenic traits, its progression [40,100,101,102], subtle microevolution [103,104,105,106,107], and metastasis [73,74,75,108,109,110,111]. To overcome these challenges, therapeutic agents for vascular normalization [112] and degradation of ECM components—such as hyaluronidases [113], collagens [114], and LOX inhibition [115]—have been developed to alleviate intratumoral pressure and improve perfusion transport of therapeutic agents and immune cell infiltration [116].

### 2.2. Immunosuppressive Chemokines: The Invisible Barrier against CAR T Infiltration

The recruitment of CAR T cells to the TME, upon systemic or regional administration, depends largely on efficient chemotactic migration. Without biochemical hints, the probability that CAR T cells localize and migrate to a solid tumor would be low. Within a TME, the local accumulation of suppressive chemokines and cytokines—such as CXCL12 [117,118], TGFβ [63,119], IL-10 [120,121,122], IL-4 [123], IL-35 [124,125] and other factors (e.g., reactive oxygen species (ROS), lactate [126], prostaglandin E2 (PGE2), and adenosine [127])—constitute a first major barrier of immune suppression. The ability to sense and migrate towards a tumor-specific site is a critical prerequisite to immune extravasation and infiltration; however, as a physiological protective mechanism, tumors produce insufficient chemoattractant ligands for T cells. Therefore, there have been significant efforts to increase CAR T chemotaxis and extravasation that endow the local delivery of favorable chemokines and cytokines (e.g., CXCL11 [128], RANTES, and IL-15 [129]); these efforts include the use of intratumorally delivered oncolytic virus to improve CAR T cell recruitment and anti-tumor activity with resulting better survival [128,129,130]. Alternatively, there have been promising strategies that employ CAR T cells as cytokine carriers to promote infiltration and maintenance of a healthy TILs population. CAR T cells—engineered to secrete proinflammatory cytokines, IL-12 [17,131], IL-18 [132], IL-7 [133], and CCL19 [134] —have been reported to augment autocrine stimulation and potentiate paracrine signaling advantageous for survival, persistence, and recruitment of endogenous immune cells.

To enhance locomotion in the TME, CAR T cells are engineered to express receptors specific to tumor-derived chemokines. This approach has shown efficient chemotactic migration and demonstrated remarkable tumor regression in both in vitro and in vivo models. T cells transduced with a retroviral vector to express CXCR2, a receptor of chemokine CXCL1, were able to enhance direct migration toward tumor-derived chemokine [135]. In another study, CCR2—a receptor of chemokine CCL2, co-expressed with a chimeric antigen receptor targeting tumor antigen GD2 [136] (expressed on neuroblastoma) and mesothelin [137] (expressed on malignant pleural mesotheliomas)—demonstrated a significant increase (more than 10-fold) in homing and anti-tumor activity. Our group, Jin et al., has developed CD70-directed CXCR1 or CXCR2-modified CAR T cells that can co-opt IL-8 with robust antitumor activity and long-lasting immunologic memory against glioblastoma, ovarian, and pancreatic cancer xenograft models [138]. This approach has received FDA IND approval soon to be evaluated in a first clinical trial in humans [138,139]. Furthermore, CAR T cells have been designed to co-express dominant-negative receptors of factors (e.g., TGFβ [140], PD-L1 [141]) to resist immune-inhibitory signals in the TME or co-express inverted cytokine receptor [123,142] to reverse suppressive cytokine functions and stimulate anti-tumor activity. In a preclinical study for breast cancer, CAR T cells targeting MUC1 co-expressed with an inverted cytokine receptor comprised of an IL-4 exodomain linked to an IL-7 endodomain reversed inhibitory signals from IL-4 present in the TME and demonstrated durable T cells infiltration and memory formation [143].

### 2.3. Pro-Tumor Stromal and Immune Cells Are Active Contributors to Immune Suppression

Once CAR T cells invade chemorepellent and physical barriers of the TME, they encounter stromal and immune cell populations that often exhibit context-dependent functionality. Cancer-associated fibroblasts (CAFs) are present in all solid tumors and represent a major component of the reactive tumor stroma integral to the development and maintenance of a complex TME [144]. CAFs are responsible for abundant production of ECM, crosslinking enzymes, and cytokines that fortify the tumor and constitute a complex and heterogenous barrier against immune attacks [145]. CAFs are major contributors to the production of immunosuppressive cytokines, such as TGFβ, which have also been shown to interfere with T cell migration and infiltration into tumors [63,146]. CAFs have been reported to cross-present antigen and kill CD8+T cells in an antigen-dependent manner via PD-L2 and FASL expression [147] and recruit other inhibitory immune cells to participate in suppressing anti-tumor function [61]. Direct immunotherapeutic attenuation of CAF activity shows promising improvement of T cell trafficking and infiltration [148]. Kakarla et al. developed CAR T cells targeting fibroblast activation protein (FAP), demonstrating a significant reduction in FAP-positive stromal cells and tumor growth in murine models. A combined application of FAP-CAR T and tumor-antigen CAR T cells resulted in anti-tumor activity superior to treatment with either CAR T alone [149]. Despite generally being known for pro-tumor function, the role of CAFs in tumor progression is not totally understood. Depletion of CAFs accelerated pancreatic ductal adenocarcinoma progression and reduced survival in transgenic mice models [150]. Although each cellular component of a TME exhibits a supportive role in tumor progression, their existing anti-tumor functions further complicate therapeutic strategies.

Tumor initiation and progression lead to inflammatory reactions that trigger recruitment and repair mechanisms by innate and adaptive immune response [151]. In the complex TME, cancer, stromal, and immune cell populations continuously evolve. The pro-tumor immune subsets within the TME generally comprise tumor-associated macrophages (TAM) [56,152], myeloid-derived suppressor cells (MDSCs), tumor-associated neutrophils (TAN), and regulatory T cells (Treg) [54,62,153]. Macrophages and dendritic cells (DCs) are important myeloid cells of the innate immune system. TAM often adopt an M2- macrophage phenotype [154] which is anti-inflammatory and pro-tumorigenic [155,156]. TAMs secrete CCL2, which recruits and activates additional TAMs. M2 TAMs have revealed pro-tumor functions and immune suppressive effects via secretion of epidermal and angiogenic factors, IL10 and TGFβ [157]. CAR T cells targeting M2-like TAMs in murine ovarian carcinoma, colon adenocarcinoma, and melanoma models reprogrammed the TME with enrichment of pro-inflammatory monocytes and activated CD8+T cells [158].

MDSCs, a heterogeneous population of immature myeloid cells, are recruited into the tumor by various chemokines CCL1, CCL2, CCL5, or CXCL5 [153,159]. MDSCs can be divided into two major subtypes: granulocytic and monocytic. Granulocytic MDSCs secrete reactive oxygen species (ROS), whereas monocytic MDSCs produce increased levels of nitric oxide derivatives resulting in decreased T cell immune responses [60,160]. A MUC1-directed CAR T cell approach concomitantly targeting tumor necrosis factor-related apoptosis-inducing ligand receptor 2 (TR2) expressed on MDSCs demonstrated enhanced antitumor tumor activity in breast cancers enriched with MDSCs and TME remodeling [161]. In addition, there is a growing body of data on the anti-antitumor immunity of TANs. The role of TANs seems to vary based on the type of solid tumors [162]. It has been described that TANs recruit MDSCs, TAMs, and Tregs to TME through secretion of IL-4, IL-10, arginine-1, and ROS which have inhibitory effects on cytotoxic T cells [162].

Tregs are cells born in the thymus and express CD4+ FoxP3. Tregs within the TME can act antagonistically to effector T cells by secreting a number of cytokines (e.g., IL10, IL35, TGFβ), upregulating cytotoxic T lymphocyte antigen 4 (CTLA4), and inhibiting CD80/CD86 co-stimulatory pathways, by competing for binding of IL-2 [163]. An agonist antibody specific against glucocorticoid-induced TNFR-related receptor (GITR) decreased Treg mediated immunosuppression which correlated with augmented anti-glioblastoma immune response [164]. Once in the TME, CAR T cells must overcome checkpoint inhibitory signals. Checkpoints (e.g., PD-L1, GITR) are physiological brakes that prevent autoimmunity [158,159,160]. However, cancer cells abuse such mechanisms to avoid immune surveillance, and the complex TME further supports cancer cells by employing multifactorial and reciprocal pathways advantageous to immune suppression. For instance, IFNγ release during CAR T cell activation and killing of tumor cells induces upregulation of immune checkpoint proteins (e.g., PD-L1) which attenuate the anti-tumor function of cytotoxic T cells [165]. On the other hand, cancer-associated cells can be reprogrammed to target cancer cells. Reinhard et al. revealed nanoparticles delivering RNA encoding for claudin 6—a developmental antigen—can transduce antigen presenting cells to express the immunological targets and enhance CAR T expansion and tumor regression in a mouse model [166].

### 2.4. Physical Confinement and Mechanical Properties of Cell Nucleus Modulate T Cell Locomotion

T cell locomotion within the TME is an important aspect of immuno-oncology and has been extensively studied [50,51,167,168]. T cells are capable of dynamically adapting to various modes of migration depending on ECM composition within the TME [50,168,169]. However, the mechanism behind this adaptation has not been clearly explained. A better understanding of T cell migration will help enhance CAR T trafficking to solid tumors. The modes of cell migration are often regulated by the density of binding sites on substrate [170], proteolytic activity [171], actomyosin contractility [50,172], microtubule stability [51], and geometrical and mechanical properties of the confined surroundings [51,53,167,169,173]. T cells are fast migrators, for they need rapid scanning abilities to carry out efficient immunological response. Two-photon microscopy imaging of intact mouse lymph nodes revealed that T cells can migrate at an average speed of 10 µm min^−1^ and peak at 25 µm min^−1^ [174]. Unlike cancer and stromal cells that actively secrete protease enzymes to degrade the ECM [59] and migrate via focal adhesion mediated attachment, T cells do not need mature focal adhesions and prefer to explore the TME via paths of least resistance [168,175,176]. In other words, leukocytes probe their surroundings and selectively squeeze through accessible pores without proteolytically breaking down ECM to create paths for migration. Although capable of integrin-dependent migration, T cells only form short-lived adhesion complexes to facilitate rapid detachment and engagement onto the next location [177]. The most common mode of T cell migration is amoeboid which employs coordinated membrane extrusion, cell contractility, and contact-induced traction in confined spaces to move forward [168,177]. Amoeboid migration is critically dependent on cortical contractility regulated via the Rho/ROCK pathway and myosin II activity [50]. It has been reported that inducing actomyosin contractility by increasing myosin II or activation of the Rho/ROCK pathway is sufficient to transform cell mode of migration into amoeboid [172,178,179]. Besides cell contractility, proper geometrical confinement is an essential determinant of amoeboid migration [169]. T cells protrude oscillatory leading-edge membrane blebs into narrow pores, probe for potential paths, and generate contraction-mediated retrograde actin flow beneath the cell membranes to transmit equal and opposite frictional forces onto the substrate to drive the cell forward [50,168].

The compliance of the cells can better facilitate constricted migration [55]. The deformability of a cell depends significantly on the mechanical property of its nucleus [180,181]. During transendothelial migration through tight spaces, CAR T may be subjected to nuclear rupture which could severely impact cell function [182]. Major determinants of nuclear stiffness are lamin A/C content and chromatin decondensation which are mechanically regulated by force transmission between cell–cell and cell–matrix interaction via cytoskeleton and LINC complex [70]. Lamin A is known to be responsible for the viscous portion of the nucleus which leads to plastic deformation post-migration, whereas lamin B is responsible for the elastic portion promoting shape recovery after deformation [183,184]. Although high lamin A expression impedes migration through small pores (<3 µm in dia.), it promotes cell survival against stress-induced apoptosis by upregulation of DNA damage repair protein, HSP90 [185,186].

Furthermore, cells are well-known mechanosensors [36,70,187,188,189,190,191] and can alter their mechanical properties and mode of migration according to the stiffness of local surroundings [51,55,57,58]. On a soft substrate, lamin A/C phosphorylation is increased; whereas on stiff substrate, myosin II-generated tension promotes dephosphorylation and stabilization of lamin A/C levels which in turn enhance nuclear stiffness [192]. Moreover, mechanotransduction is a key regulator in T cell activation [193,194] and cytotoxic activity [191]. In the context of T cell migration, the mechanical stiffness of the substrate is important for durotaxis and contact guidance-directed locomotion in 3D [51]. T cells apply traction forces (100 pN) via TCR to physically probe the rigidity of their surroundings [189] and migrate along with the aligned ECM fibers [195]. Although matrix optimal confinement and alignment could induce rapid T cell amoeboid migration, there is a limit to which cells are physically trapped and suppressed. For instance, high-density collagen deposition and crosslinking have been shown to suppress proliferation and anti-tumor activity of infiltrating CD8+ T cells in triple-negative breast cancer tissue explants [84]. Another study shows that a densely packed and oriented stromal fibers at the perivascular niche and around the tumor impose a significant challenge to T cell infiltration [52]. Recently, Caruana et al. developed CAR T cells expressing heparinase to degrade HSPGs and promote tumor infiltration. In xenografted mouse models, the strategy improved T cell capability to degrade ECM, infiltrate, and carry out antitumor activity [47].

## 3. Preclinical Models for Trafficking and Infiltration

In this section, preclinical approaches aimed to improve CAR T cell trafficking and survival are summarized. While most inhibitory barriers of the TME mentioned in the previous section have been considered and targeted, much work remains to be studied for better translation to clinical outcomes (Table 1).

### 3.1. In Vivo Models

In vivo murine models have provided a great understanding of anti-tumor CAR T cell activity [139]. The preclinical murine models can be divided into two major groups: immunocompromised and immunocompetent models. Frequently utilized models encompass immunosuppressed, NSG (e.g., NOD SCID) mice where human tumor cells and adoptively transferred immune cells can survive and persist in the host. However, these immune-suppressed models lack autologous immune cells, thus providing unreliable representation of a TME [202]. To overcome such limitations, immunodeficient mice are transplanted with human CD34+ hematopoietic stem cells (HSCs) to reconstitute with human immune components. Ideally, autologous patient-derived tumor xenografts (humanized PDXs) can be implanted in those humanized murine models to closely recapitulate the interactions between the TME-associated stromal and immune components and immunotherapeutic interventions (e.g., CAR T cells) seen in human patients [139,203,204]. Given the low rate of tumor engraftment in autologous systems, there have been multiple approaches to improve tumor implantation such as using: (1) closely HLA matched HSCs, which might represent a potential confounder of allogeneic immunoreactivity; (2) expanded autologous-infiltrating T cells; or (3) engineering HSCs to secrete cytokines that favorably condition the host environment for immune cells [205,206,207,208].

In immunocompetent systems, the TME can be studied as transplantable models where murine tumor cell lines harbor multiple evolving mutations which does not follow the sequential mutational evolution of human cancers [202,209]. The emergence of genetically engineered mouse models (GEMMs) has gotten us closer to what actually happens in tumorigenesis, tumor progression, and metastasis in human patients. Particularly, advanced GEMMs with patient-relevant mutations are engineered to model clinically pertinent tumors that might behave differently to immunotherapies compared with their null counterparts (e.g., Trp53 hotspot mutations, RING-specific BRCA1 mutations) [210,211,212,213]. GEMM models allow for a more accurate mirroring of human tumor heterogeneity in an immunocompetent environment where immunotherapies (e.g., CAR T cells) can be studied more faithfully [214,215,216]. Using these models, CAR T cell migration can be evaluated by a variety of imaging techniques including reporter gene-transfected CAR T cells (e.g., luciferase), in vivo imaging using two-photon intravital microscopy techniques, and magnetic particle imaging (MPI) that leverages the use of nanoparticle cell tracers [138,139,217,218]. These immunocompetent murine models however are not perfect replicates of human immune biology.

There is increasing interest in preclinical large animal models, such canine models and oncopig models. In comparative oncology that studies animal tumor models that develop cancers spontaneously, pet dog models are gaining significant attraction as they are one step closer to real world cancers given not only clinical and molecular similarities but also patient heterogeneity. A number of trials using different immunotherapy approaches—including CAR T cells—are used to treat HER2+ osteosarcoma, B7-H3+ sarcomas, and gliomas [4,219]. However, the paucity of patient recruitment similarly seen in human trials might need more expeditious cancer models. Genetically engineered pigs (oncopigs or OCMs) represent an exciting approach to study large animal models with substantial anatomic, genomic, immunologic, and tumor micro niche similarities; patient heterogeneity; and, additionally, a cohort of oncopigs could potentially be readily available for correlative in vivo studies simultaneously carried out with human clinical trials [220]. The oncopigs can be genetically modified to express patient-relevant mutations, such as the model that harbors a Cre-recombinase induced expression of KRAS and TP53 mutations, that have been identified in many human cancers [221]. The immune profiling of pigs demonstrates the high correlation between their innate and adaptive immune systems as well as the intratumoral T cell milieu and the immune signature of human cancers [222,223,224,225,226]. Further characterization of the whole immune repertoire of tumors developed in oncopigs will allow better understanding of the role of TME and its crosstalk with CAR T cells for better optimization of adoptive T cell therapies. The goal of this review however will not focus on covering the novelties and advantages of genetically modified small and large animal cancer models in detail.

Despite cell trafficking being one of the major limitations of CAR T cell activity, most of the clinical trials focused largely on targeting single tumor-specific immune targets. We have identified relevant clinical trials proposed to improve CAR T cell trafficking by locoregional delivery of immune cells, genetically modified to express chemokine receptors (e.g., CCR4 and CXCR5) or to reprogram the TME. Locoregional delivery of CAR T cells is an attempt to secure direct delivery of CAR T cells bypassing peripheral blood circulation and perhaps the blood–brain barrier as in the case of brain tumors. Our group has taken advantage of IL-8 tumor secretion and genetically engineered CD70-directed CAR T cells to constitutively expressed IL-8 receptor to be tested in a first-in-human trial (NCT05353530) [138]. Furthermore, novel approaches are under investigation to positively reprogram the TME to enhance CAR T cell migration into tumor niche (Table 2) [227]. 

To complement these models, we discuss three-dimensional in vitro models to evaluate cell–cell interaction dynamics, which include proliferation upon effector–target engagement, duration of interaction, tumor escape kinetics, exhaustion, and rate of killing. These technologies can potentially be considered for use as theranostic tools for biomarker identification, response to immunotherapies, and co-clinical trial models performed simultaneously to human clinical trials for treatment decision evaluation.

### 3.2. Three-Dimensional (3D) Supports and In Vitro Tumor Models

Fundamental research in biology has been trapped by the engineering blueprints of ubiquitous 2D infrastructure, where cells are constrained to grow in a monolayer as copies of their original biology in a plastic dish. In conventional T cell killing assays, CAR T cells are directly cocultured with cancer cells in a 2D monolayer. Therefore, the lack of 3D support and physical barriers in these models ignores the need for trafficking which is a crucial challenge of CAR T therapy for solid tumors. By doing so, T cell locomotion and cytotoxic function against cancer cells are erroneously simplified. Lack of success in early phase studies is often due to an absence of biological response correlates and the inaccurate representation of a TME in oversimplified in vitro models [228,229,230,231]. 3D in vitro models can better recapitulate a TME from distinct cell types, presence of surrounding ECM, cellular trafficking, and drug sensitivity [232]. Furthermore, cells cultured in 3D may alter gene expression, leading to distinct phenotypic traits [233,234] as compared to 2D monolayers. Sureban et al. demonstrated that depending on the clonogenic capacity, surface markers of colorectal cancer cell lines grown as spheres in 3D can be different than those grown on 2D monolayer, resulting in distinct CAR T mediated cytotoxicity and associated IFNγ release [235]. Due to advancements in biotechnologies, various 3D in vitro models and platforms have been invented and applied to investigate CAR T cell motility, antigen specificity, and killing efficacy [236,237,238,239]. In this section, we review existing 3D in vitro models for preclinical assessment of CAR T therapy. These models are comprised of spheroids [236], organoids [239], or patient-derived explants [240,241] which are cultured in liquid media [237,238], hydrogels [83,237,242,243,244], or microgel platforms [245,246,247]. The existing preclinical 3D models and their associated in vitro platforms are valuable tools to advance CAR T therapy for solid tumors; however, as models, they are not meant to be the perfect representation of in vivo conditions. Understanding the advantages and disadvantages of each model will help select optimal approaches for each application.

### 3.3. Common Three-Dimensional Support Materials for In Vitro Assays

Hydrogels have been empowered as materials of choice for 3D cell culture platforms [83,237,242,243,244]. Hydrogels can be classified into naturally derived, synthetic, and hybrid. Natural hydrogels include collagen [28,111,248], Matrigel^TM^ [242,249], fibrin [250,251], and alginate, [101,252,253]. Alternatively, synthetic hydrogel counterparts are commonly comprised of polyacrylamide (PAAm) [254] and polyethylene glycol (PEG) [255]. The major advantages of natural hydrogels are their cytological compatibility and the presence of intrinsic cell adhesion molecules. Some natural hydrogels have tunable mechanical properties (e.g., collagen, fibrin) and allow closer recapitulation of different tissues [250,256]. However, modulating polymer concentration of natural hydrogels is often compromised by the expense of available cell adhesion density [244]. Alternatively, the mechanical properties of synthetic hydrogels simply require modification of monomer to crosslinker ratio or polymer contents independent of ligand density for cell adhesion [257,258,259]. Natural hydrogels often contain great batch-to-batch variability and ill-defined protein composition [243,260], making reproducibility and consistency challenging [243,261,262]. For PEG or PAAm synthetic hydrogels, the polymer content, mesh size, and elastic modulus can be chemically predetermined and synthesized with trivial variation between batches [259,263]. Furthermore, natural hydrogels are either particularly sensitive to protease enzymes (e.g., fibrin) or nondegradable (e.g., alginate), which present limitations for long-term cell culture studies [244]. On the other hand, PEG crosslinkers are either non- or MMP-degradable, offering a relevant ECM synthetic material for 3D cell culture studies—including migration, differentiation, angiogenesis, and organoid assembly [243,264,265].

The microgel platforms represent alternatives to hydrogels. Microgels have been increasingly applied in biomedical research. More comprehensive reviews of microgel platforms have been reported elsewhere [246,247]. In brief, microgels belong to a special class of materials that unites all three major classes of colloids: rigid particles, flexible macromolecules, and surfactants [245,246,247]. Microgels are aqueous and crosslinked macromolecular networks of hydrogel colloidal particles swollen by solvents. Like hydrogels, the monomer-to-crosslinker ratio dictates the elastic modulus and mesh size of the microgels, thereby determining their colloidal or macromolecule character [245,246]. External stimuli—such as temperature, ionic strength, pH, pressure, and light—could alter the mass exchange of the microgel with the surroundings thereby changing their volume, shape, and mechanical properties [246,247]. The properties of microgels can be optimized to specific applications in the synthesis process with proper selection of building blocks and reaction sequence [246]. Microgel platforms from different ensembles of (e.g., PEG [264], PAAm [266], hyaluronic acids [267], or gelatin methacryloyl (GelMA) microbeads [268]) have been valuable tools for drug delivery [269,270], wound healing [264], and mechanotransduction studies [271].

### 3.4. Tumor Spheroids

Tumor spheroid models are the simplest 3D in vitro models that can be generated by methods of cell aggregation (e.g., hanging drop [272], ultralow attachment plates [273]). These straightforward methods enable great control over sample size and the number of cells for each spheroid. The tumor spheroids can be cultured in liquid media or embedded in a hydrogel [274]. Platforms (e.g., hydrogels or microgels) that support cells in 3D can better recapitulate the physical and biochemical constraints to better study the trafficking of CAR T in solid tumors. Wallstable et al. applied a small intestinal submucosa and mucosa (SISmuc) hydrogel platform—a collagen matrix with an intact basement membrane derived from decellularized porcine jejunum—to demonstrate CAR T efficient migration into the ECM, infiltration into the tumor, and elimination of tumor cells [237]. In another study, Ando et al. developed a microfluidic platform encasing a GelMA to study the role of hypoxia on immune-tumor interactions, which revealed that CAR T cell-mediated killing is dependent on oxygen gradients [238]. Similarly, Grunewald et al. applied 3D bioprinting to construct 3D TMEs [275] allowing for detection and quantification of L1 cell adhesion molecule (L1CAM). CAR T infiltration in this bioprinted 3D (neuroblastoma) model showed activation superior to 2D assays [275]. These tumor spheroid models resemble solid TMEs, which is useful for investigations of immune-tumor interactions, tumor invasion, or drug resistance [276]. However, the spheroid models are formed using immortalized cell lines, making them less recapitulative of in vivo tumors than organoids and patient-derived explants.

### 3.5. Tumor Organoids

Organoids are alternative 3D in vitro models often cultured in a 3D hydrogel support platform. The organoids are cultured from embryonic, adult, or induced pluripotent stem cells [276,277]. Tumor organoids are generated from patient-derived tumor explants by means of tissue dissociation, enzyme digestion, collection of single cells, and embedment culture in hydrogels (Matrigel^TM^). The isolated cells are cultured in particular growth factors such as Wnt activators (Wnt3a and R-spondin), exogenous receptor tyrosine kinase ligands (epidermal and fibroblast growth factors), and bone morphogenetic proteins antagonist noggin, among others [239,276]. Not only can the organoids recapitulate original tissue histologically and genetically, but they also have the ability to self-organize, differentiate, and function in response to mechanical and chemical cues [276,277]. Organoids are widely cultured in 3D in various platforms such as static ECM-embedment culture [237], microfluidic devices [248], and 3D bioprinting [275]. In one study, co-culture of CAR-engineered natural killer cells (EPCAM-CAR NK-92) and patient-derived colon organoids on the Matrigel matrix demonstrated an increase in immune cell migration and cytotoxicity against cancer cells [239]. Although the tumor organoid models have gained popularity in basic cancer research, the initial tissue dissociation, enzyme digestion, potential clonal selection, and lengthy production time are major disadvantages of the models. The process of tissue digestion removes natural ECM structure and eliminates endogenous immune and stromal cell components. The subsequent culture of isolated cancer cells in Matrigel^TM^ containing exogenous and ill-defined growth factors potentiates significant modification of the original tumor organization and cell types.

### 3.6. Tumor Explants

In 1985, Robert Hoffman et al. pioneered the histoculture studies of tumor explants. The study showed that different cell types within the cultured tissue slices can grow, organize, differentiate, and maintain in vivo properties [278]. Despite its great potential, the models have not received much attention for the last two decades. The difficulty to maintain these tissue explants ex vivo, coupled with the straightforward application of cellular spheroids and the increasing popularity of organoid models, may have overshadowed the application of tumor explants. In recent years, the tumor explant models have gained traction due to advancements in cell culture platforms [11,279] and identifications of novel media compositions. Unlike spheroid and organoid models, resected tumor explants are directly cultured in well-studied media or 3D supported (e.g., hydrogels, microgels) platforms without enzyme digestion and cells isolation process, leading to better preservation of heterogeneity of the original tumor [241,279,280]. In one study, Jacob et al. described a procedure of generation and biobanking of glioblastoma organoid (GBO)-like explants from ex vivo culture [240]. The GBO explants were cultured without tissue dissociation, cell selection, or Matrigel matrix [240]. The resected and minced microexplants were cultured in a specialized culture medium on an orbital shaker which resulted in organoid-like phenotypes with a proliferation rate similar to that of parent tumors [240]. The GBO can be passaged and cryopreserved efficiently allowing repeatability of experiments. Furthermore, the heterogeneity of the GBO model was maintained as demonstrated by the expression of endogenous mutant proteins, epidermal growth factor receptor variant 3 (EGFRvIII), commonly expressed in glioblastomas. Co-culture of EGFRvIII specific-CAR T with GBO samples demonstrated immune cell proliferation and activation with antigen-specific tumor cell killing [240]. The significant roles of the TME and its heterogeneity highlight the need for these complimentary 3D in vitro models and platforms to better evaluate cancer biology and immunological trafficking.

## 4. Conclusions

This review highlights the significant difficulties imposed by biochemical and physical barriers on CAR T trafficking and infiltration in the TME. Despite a better understanding of tumor biology and promising new treatment options, CAR T therapy remains largely untapped in solid tumors. The shortcoming of therapeutic success highlights the need for advanced preclinical models with the well-preserved complex TME and the associated immune suppressive components. The mechanistic studies of CAR T trafficking and interaction with solid cancers are difficult to carry out solely in animal models which are fundamentally different from human biology. Advanced 3D in vitro models provide a complementary approach to enable investigations at the single-cell level, thereby providing better insights to CAR T cell design, activity, and therapeutic applications.

## Figures and Tables

**Table 1:** Preclinical CAR T cell approaches to improve trafficking.

Reference	Tumor	TME	Appproach	Outcome(s)
[196]	B16 melanoma	Vasculature	VEFGR-2 CAR T cells in combination with anti-VEFG-A ligand antibody	Combination of anti-VEFG-A ligand to overcome competition enhances CAR T cell activity
[197]	Murine breast carcinoma	ECM	Engineered macrophages to HER2 CAR T cells and production of metalloproteases as byproduct of activation	In addition to controlling HER2+ tumors, this approach decreased collagen deposition and facilitated T cell infiltration
[47]	Stroma-rich solid tumors	ECM	CAR T cells expressing enzyme heparanase (HPSE)	The expression of HPSE improves CAR T cell infiltration through degradation of the ECM
[198]	Breast, colon, melanoma, fibrosarcoma	ECM	Fibroblast activated protein targeted CAR T cells (FAP-CAR T cells)	Toxicity and off-target effects in bone marrow killed mouse models and therapy showed limited antitumor results
[148]	Carcinomas	ECM	FAP-CAR T cells	Decreased tumor density and reduced autochthonous pancreatic cancer growth
[149]	Non-small cell lung carcinoma	ECM	FAP-CAR T cells	Reduction in FAP positive stroma and improved antitumor killing alone or when paired with anti-EpHA2 CAR T cells
[199]	Hepatocellular carcinoma (HCC)	Migration	CXCR2-expressing CAR T cells	Improved T cell migration and accumulation at the tumor site compared to control
[200]	Solid tumors	Hypoxemia	CD19 and B cell maturation antigen (BCMA) CAR T cells at normoxic and hypoxic oxygen levels	Hypoxic conditions attenuated CAR T cell expansion, differentiation to CD8 T cells, and cytokine production
[201]	Solid tumor	Hypoxemia	CAR T cells were transcriptionally paired to hypoxia response elements (HREs) including hypoxia-inducible-1 factor-alpha (HIF1α)	Hypoxia-induced CAR T cells showed improved antitumor activity in hypoxia compared with normoxia
[166]	Solid tumor	Immune cells	Delivery of developmental antigen-encoding RNA via nanoparticles	RNA loaded nanoparticles enhanced expansion and activity of CAR T cells in claudin-expression solid tumors

**Table 2:** Human clinical trials with strategies to enhance CAR T trafficking.

NCT	Title	Phase	Author(s)	Disease(s)	Approach	Status
NCT04185038	Study of B7-H3-Specific CAR T Cell Locoregional Immunotherapy for Diffuse Intrinsic Pontine Glioma/Diffuse Midline Glioma and Recurrent or Refractory Pediatric Central Nervous System Tumors	I	Vitanza et al.	DIPG/recurrent pediatric CNS tumors	Locoregional delivery targeting B7-H3	Recruiting
NCT03696030	HER2-CAR T Cells in Treating Patients with Recurrent Brain or Leptomeningeal Metastases	I	Pornow et al.	CNS metastasis	Intraventricular delivery of HER2 CAR T cells for CNS metastasis from HER2+ tumors.	Recruiting
NCT03638167	EGFR806-specific CAR T Cell Locoregional Immunotherapy for EGFR-positive Recurrent or Refractory Pediatric CNS Tumors	I	Gust et al.	EGFR-positive recurrent or refractory pediatric CNS tumors	Locoregional delivery targeting EGFR806	Recruiting
NCT03283631	Intracerebral EGFR-vIII CAR-T Cells for Recurrent GBM (INTERCEPT)	I	Landi et al.	Recurrent GBM	Intracerebral EGFR-vIII CAR T cells	Terminated. Patient enrollment was halted.
NCT04153799	Study of CXCR5 Modified EGFE Chimeric Antigen Receptor Autologous T cells in EGFR-Positive Patients with Advanced Non-Small Cell Lung Cancer	I	Zhang et al.	Advanced non-small-cell lung cancer	CXCR5 modified CAR T cells targeting EGFR	Recruiting
NCT03602157	Study of CAR-T Cells Expressing CD30 and CCR4 for r/r CD30+ HL and CTCL	I	Grover et al.	Relapsed and recurrent Hodgkin lymphoma and cutaneous T cell lymphoma	CCR4 modified CAR T cells targeting CD30	Recruiting
NCT05081479	A Study of N-Acetylcysteine (N-AC) in People Receiving CAR T cell Therapy for Lymphoma	I	Batlevi et al.	B cell lymphoma	Reduce tumor reactive oxygen species to condition lymphoma TME for CD19 CAR T cells	Recruiting
NCT04976218	TGFβR-KO CAR-EGFR T Cells in Previously Treated Advanced EGFR-positive Solid Tumors	I	Han et al.	EGFR-positive solid tumors	CAR T cells resistant to TGFβ receptor	Recruiting
NCT03740256	Binary Oncolytic Adenovirus in Combination with HER2-Specific Autologous CAR VST, Advanced HER2 Positive Solid Tumors (VISTA)	I	Wang et al.	Advanced HER2+ solid tumors	Oncolytic adenovirus delivers PDL1 blocking mini antibody to enhance CAR T cell killing	Recruiting
NCT04381741	CD19 CAR-T Expressing IL7 and CCL19 Combined with PD1 mAb for Relapsed or Refractory Diffuse Large B Cell Lymphoma (CICPD)	I	Qian and Liu et al.	Recurrent or relapsed diffuse large B cell lymphoma	CAR T cells are potentiated with co-expression of IL-7 and CCL19 to better migrate into the TME and enhanced T cell fitness. This approach is also combined with PD1 blocking antibody.	Recruiting
NCT03545815	Study of CRISPR-Cas9 Mediated PD-1 and TCR Gene-Knocked Out Mesothelin-Directed CAR-T Cells in Patients with Mesothelin Positive Multiple Solid Tumors	I	Han et al.	Mesothelin-directed CAR T cells	CAR T cells have PD1 TCR receptor knocked out to enhance their activity	Recruiting
NCT02706405	JCAR014 and Durvalumab in Treating Patients with Relapsed or Refractory B-Cell Non-Hodgkin Lymphoma	1b	Gauthier et al.	B cell non-Hodgkin lymphoma	PDL1 blocking antibody to improved CD19 CAR T cells	Terminated due to slow accrual
NCT03070327	BCMA Targeted CAR T Cells with or without Lenalidomide for the Treatment of Multiple Myeloma	I	Mailankody et al.	Multiple myeloma	Lenalidomide has been shown to inhibit regulatory T cells and activate CD8 T cells	Active, not recruiting

## Data Availability

Not applicable.
